# Crystallization Scale Preparation of a Stable GPCR Signaling Complex between Constitutively Active Rhodopsin and G-Protein

**DOI:** 10.1371/journal.pone.0098714

**Published:** 2014-06-30

**Authors:** Shoji Maeda, Dawei Sun, Ankita Singhal, Marcello Foggetta, Georg Schmid, Joerg Standfuss, Michael Hennig, Roger J. P. Dawson, Dmitry B. Veprintsev, Gebhard F. X. Schertler

**Affiliations:** 1 Laboratory of Biomolecular Research, Paul Scherrer Institut, Villigen, Switzerland and Department of Biology, ETH Zurich, Zurich, Switzerland; 2 pRED Pharma Research and Early Development, Small Molecule Research, Discovery Technologies, F. Hoffmann-La Roche Ltd, Basel, Switzerland; Universitat Politècnica de Catalunya, Spain

## Abstract

The activation of the G-protein transducin (Gt) by rhodopsin (Rho) has been intensively studied for several decades. It is the best understood example of GPCR activation mechanism and serves as a template for other GPCRs. The structure of the Rho/G protein complex, which is transiently formed during the signaling reaction, is of particular interest. It can help understanding the molecular details of how retinal isomerization leads to the G protein activation, as well as shed some light on how GPCR recognizes its cognate G protein. The native Rho/Gt complex isolated from bovine retina suffers from low stability and loss of the retinal ligand. Recently, we reported that constitutively active mutant of rhodopsin E113Q forms a Rho/Gt complex that is stable in detergent solution. Here, we introduce methods for a large scale preparation of the complex formed by the thermo-stabilized and constitutively active rhodopsin mutant N2C/M257Y/D282C(RhoM257Y) and the native Gt purified from bovine retinas. We demonstrate that the light-activated rhodopsin in this complex contains a covalently bound unprotonated retinal and therefore corresponds to the active metarhodopin II state; that the isolated complex is active and dissociates upon addition of GTPγS; and that the stoichiometry corresponds to a 1∶1 molar ratio of rhodopsin to the heterotrimeric G-protein. And finally, we show that the rhodopsin also forms stable complex with Gi. This complex has significantly higher thermostability than RhoM257Y/Gt complex and is resistant to a variety of detergents. Overall, our data suggest that the RhoM257Y/Gi complex is an ideal target for future structural and mechanistic studies of signaling in the visual system.

## Introduction

Intercellular signaling is essential for complex biological processes in higher animals, such as differentiation, immune response, metabolic regulation, and neural activity. The largest group of proteins involved in these processes is the G-protein-coupled receptors (GPCRs) that transmit the signal across the cellular membrane. Despite their functional and ligand diversity, all GPCRs share a seven alpha-helical transmembrane architecture and presumably transduce the activation signal by a common mechanism via a heterotrimeric guanine nucleotide-binding protein (G-protein). GPCR malfunction is often associated with pathological outcomes [1], [2], [3] and hence, these receptors constitute an important pharmaceutical target, with almost 30% of currently prescribed drugs acting through the GPCR family of proteins [4]. Although structure determination of GPCR has been remarkably accelerated in recent years due to the innovative protein engineering [5], [6], [7], novel crystallization techniques [8] and crystallography methods [9], [10], only a few structures are crystallized in the active conformation [11], [12], [13], [14], [15] and only one structure of an active GPCR/G-protein complex [16] has been determined so far. Apart from rhodopsin, structures of active GPCRs are obtained from heavily modified proteins with truncated termini/loops, fusion domains and co-crystallized antibodies. Further structures from additional receptors with minimum modifications and in complex with other types of G-proteins, arrestins and kinases will therefore be needed for a comprehensive understanding of the molecular mechanisms of GPCR signaling.

Rhodopsin is one of the most extensively studied members of the GPCR family. It works as a photoreceptor pigment protein in retinal rod cells where it senses light via covalently bound 11-*cis*-retinal that in the dark acts as a potent inverse agonist and suppresses activity of the receptor. Upon absorption of light, 11-*cis*-retinal isomerizes to the full agonist all-*trans*-retinal which in turn starts a series of conformational changes resulting in the formation of metarhodopsin-II (MetaII), the fully active species that couples with the heterotrimeric G-protein transducin (Gt). The MetaII catalyzes exchange of GDP to GTP in the Gtα subunit. Gαt and Gβγt then dissociate and diffuse to transmit the signal to downstream effectors [17].

Similar to ligand binding in other GPCRs, retinal isomerization in rhodopsin disturbes the equilibrium between different states of the receptor. In contrast to most GPCRs, rhodopsin however has an exceptionally low level of basal activity i.e. G-protein signaling in the absence of ligands [18]. The rate of spontaneous activation events in primate rod cells has been estimated to be 5.2×10^−11^ per second, corresponding to a half life of the inactive state of over 400 years [19]. Even the apoprotein opsin is 10^6^-fold less active than the active metarhodopsin II state with covalently bound agonist all-trans retinal [20]. This exceptionally low level of basal activity is required to create such a sensitive light detector as the rod cell but can be easily disturbed even by single point mutations. Such constitutively active mutations are known to shift the equilibrium towards the active form and increase the basal activity of opsin [21], [22]

In previous studies we have combined constitutively active mutants with a cysteine double mutant (N2C/D282C) that increases thermal stability but does not change retinal binding, G-protein activation, activation pathways or structure of the protein [23], [24], [25]. Of particular interest is the combination with the M257Y mutant (RhoM257Y), one of the strongest constitutively active mutants known in rhodopsin [26]. We have previously solved the crystal structure of this mutant in complex with a peptide resembling the C-terminus of the Gα protein subunit [11]. Our previous analysis of the structure suggested that the constitutive activity of this mutant stems from a stabilization of the open G-protein-binding pocket. Here we demonstrate how the RhoM257Y can be used for the purification of large quantities of complete rhodopsin/G-protein complexes for structural studies.

## Results

### Milligram scale preparation of the RhoM257Y/Gt complex

**Figure 1:**
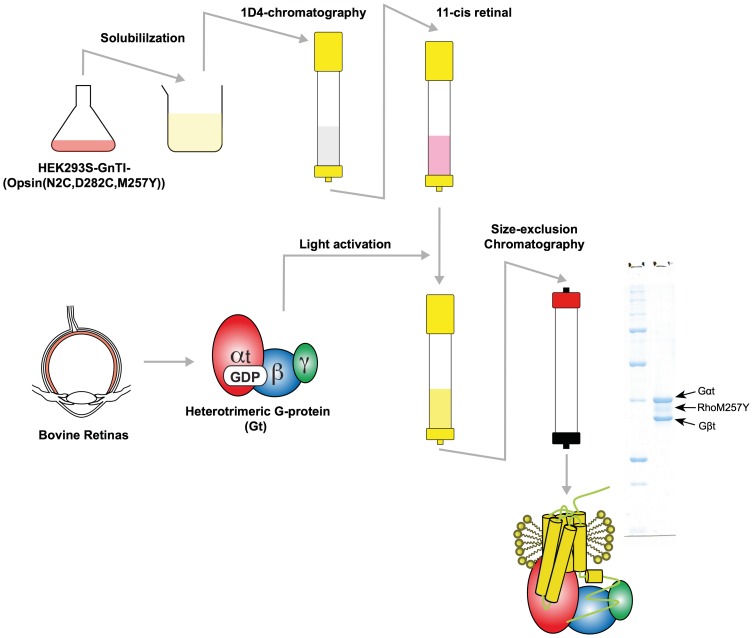
Preparation diagram of RhoM257Y/Gt. A typical 1D4 immuno-affinity purification and preparative size-exclusion chromatography of RhoM257Y/Gt from recombinant OpsinM257Y and native Gt. SDS-PAGE analysis (small inset) shows clean bands for the Gαt and Gβt subunits whereas rhodopsin runs as less sharp band due to partly heterogeneous glycosylation. The Gγt is not resolved in this gel system due to its small molecular size.

The activated rhodopsin/Gt complex was prepared by first solubilizing HEK293S-GnTI^−^ cells expressing the N2C/M257Y/D282C opsin in 1.25% DDM (w/v), centrifuging the material to remove nuclei and insoluble fractions, and applying the supernatant fraction to a 1D4-antibody immunoaffinity matrix (Figure 1) essentially as described previously [24], [27]. The immobilized opsin was then reconstituted with 11-*cis*-retinal to ground state rhodopsin while still bound to the resin, free unbound retinal was washed away and excess Gt (typically 1.5 times molar ratio of rhodopsin) was added. Complex formation was induced by isomerization of 11-*cis*-retinal to the full agonist all-*trans*-retinal using a xenon lamp with a 495 nm long-pass filter to prevent isomerization of unbound retinal. After extensive washing of the resin, rhodopsin in complex with Gt was released from the immunoaffinity matrix by incubating with the 1D4-elution peptide resembling the C-terminus of rhodopsin (TETSQVAPA). The eluted fraction was concentrated and further purified by size-exclusion chromatography to remove free components. Initially size-exclusion chromatography showed that our preparation contained a relatively large amount of free rhodopsin, supposedly due to dissociation of the complex by re-binding of GDP to Gαt. Adding apyrase, an enzyme that hydrolyzes GDP to GMP and phosphate, during the light activation improved the efficiency of the RhoM257Y/Gt complex formation by preventing re-binding of GDP. After the improvement the protein was eluted as a symmetric peak from the size exclusion column that contained all components of the RhoM257Y/Gt complex (Figure 1, small inset). A typical yield from 40 g of cell pellet and 50 retinas was 6–9 mg of RhoM257Y/Gt complex with the purity suitable for high-throughput crystallization screening.

### Spectroscopic characterization of the RhoM257Y/Gt complex

**Figure 2:**
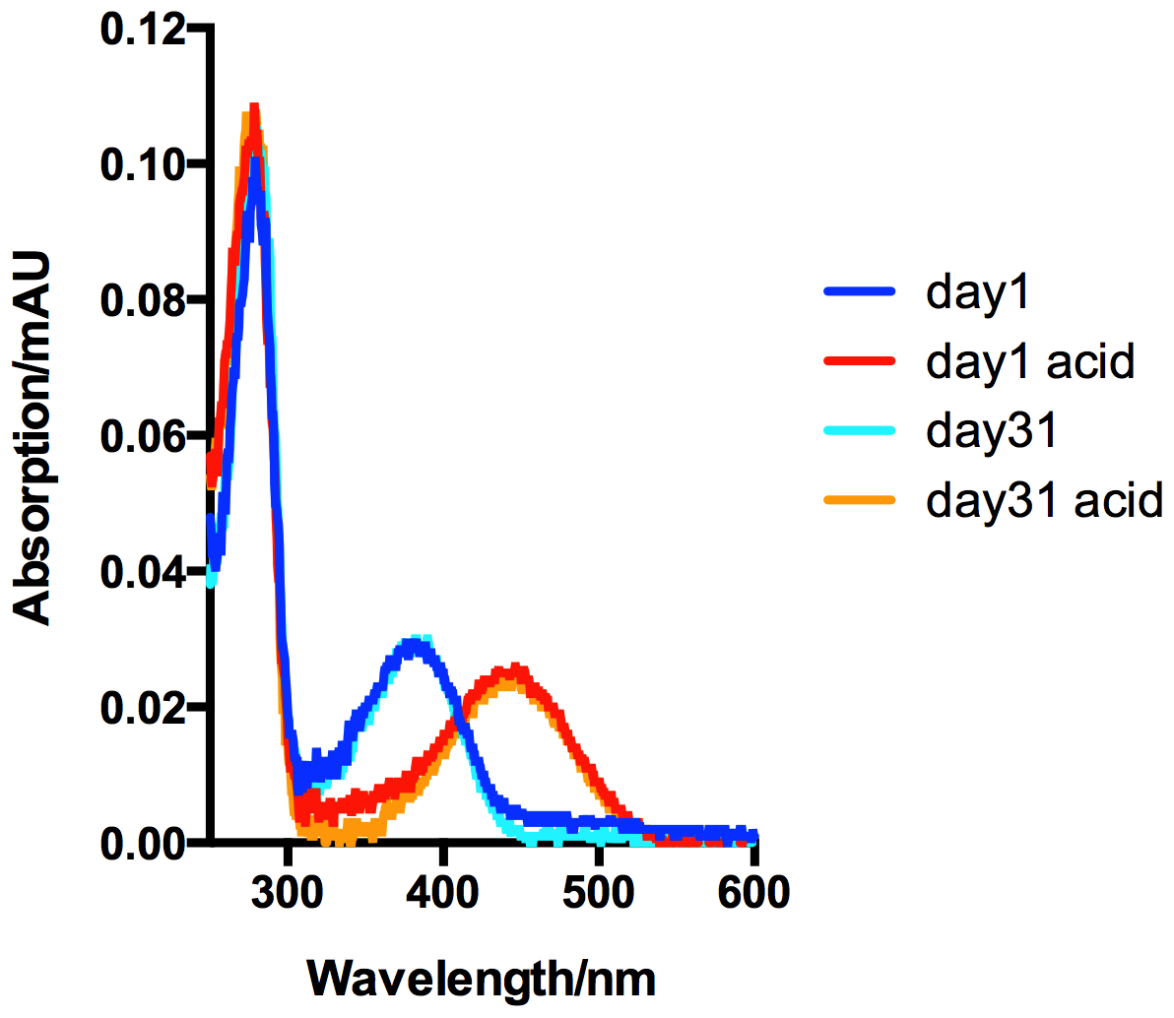
RhoM257Y/Gt complex formation evaluated by UV/VIS absorption spectroscopy. The spectrum of the purified RhoM257Y/Gt complex (blue) consists of two components, a peak at 280 nm from the protein component and a peak at 380 nm from retinal. The ratio of the two peaks for the active MetaII state is 1.6 but rises to 3.5 upon binding of the Gt protein. Based on the extinction coefficients of all components the spectrum indicates one retinal per RhoM257Y/Gt complex. Acid denaturation of the complex (red) shifts the retinal peak to 440 nm corresponding to a protonated retinylidene Schiff base. The retinal contained in our preparation is thus covalently bound to the RhoM257Y/Gt complex. Importantly even after incubation for 31 days at 4°C (cyan) the retinal is still covalently bound (orange) indicating that we prevented hydrolysis of the SB and trapped the active Rho/Gt complex under these conditions.

The photoactive ligand bound to rhodopsin is an 11-*cis*-retinal molecule covalently attached via a protonated Schiff base (SB) with K296 in TM7. Upon absorption of a photon, 11-*cis*-retinal isomerizes to all-*trans*-retinal and the proton is transferred from the SB to the retinal counterion E113 as part of a series of conformational and spectrophotometrically detectable changes leading to formation of the G-protein binding conformation MetaII [17], [28], [29], [30], [31]. The UV/VIS spectrum of the RhoM257Y/Gt complex shows two major peaks, one at 380 nm arising from unprotonated retinal (Figure 2) and one at 280 nm derived from the protein moiety. The ratio between the two peaks is 3.5 and agrees well with a single retinal per complex based on the theoretical extinction coefficients and reports in the literature [27], [32], [33]. When a purified sample of the complex was denatured by addition of acid, the 380 nm peak position was shifted to a new λ_max_ at 440 nm characteristic of a protonated retinylidene Schiff base, with little change in absorbance at 280 nm. This complete shift of the 380 nm peak under acid conditions suggests that most of the retinal is covalently bound to protein, and virtually no retinal remained free in the sample, as expected for the native Rho/Gt complex. In contrast to previously reported preparations of detergent solubilized native Rho/Gt complex, the covalently bound retinal in our mutated complex was stably trapped within the protein with no observable spectral changes after one month in detergent solution (Figure 2).

### RhoM257Y/Gt complex activity and stability

**Figure 3:**
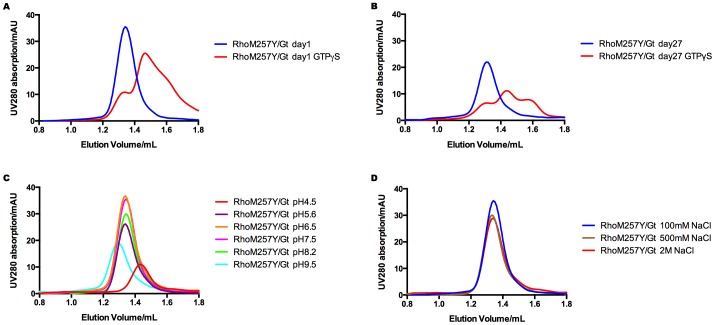
Analytical SEC of RhoM257Y/Gt. **A,B:** RhoM257Y/Gt complex retains its activity as shown by its dissociation upon incubating with GTPgS 1day after and even 27days after purification. **C,D:** RhoM257Y/Gt complex is resistant to various pH range from 5.6 to 8.2 and up to 2M NaCl salt concentration.

Besides the availability in sufficient amounts, it is critical for a successful crystallization to maintain the protein in a folded and functional state. We therefore tested the purified RhoM257Y/Gt complex for specific dissociation upon binding of the non-hydrolysable GTP analogue GTPγS. Indeed the profile obtained by analytical size exclusion chromatography showed a near complete dissociation of the complex upon specific binding of GTPγS, indicative for a high activity in the purified RhoM257Y/Gt complex (Figure 3, A). And purified RhoM257Y/Gt did not lose its activity at 4°C for 27 days after purification (Figure 3, B). Furthermore, RhoM257Y/Gt complex was resistant to wide range of pH and high salt conditions (Figure 3, C,D).

### Complex Stoichiometry

**Figure 4:**
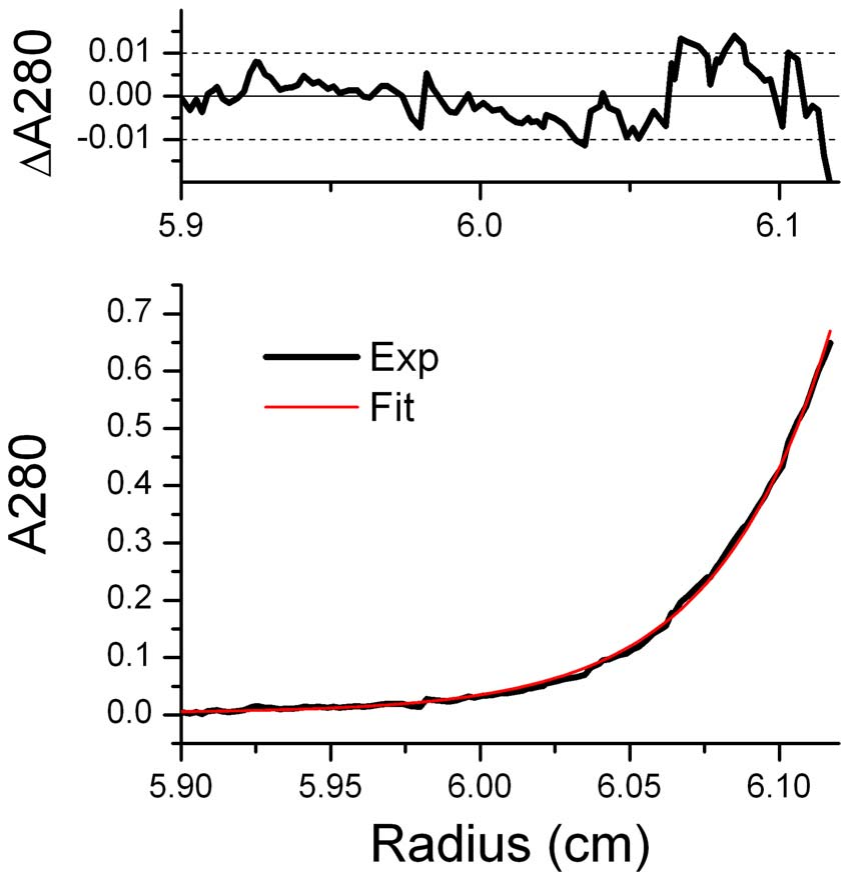
RhoM257Y/Gt complex has 1∶1 stoichiometry as shown by the equilibrium sedimentation.

Bovine rhodopsin was observed to form arrays of dimers in the native rod outer segment membrane [34], [35] and some biochemical/biophysical data suggested the formation of heteropentameric complex with Gt [36], [37], [38] at a stoichiometry of 2 rhodopsin to 1 Gt. Other data, however, suggested a stoichiometry of 1 rhodopsin to 1 Gt both in the membrane and detergent solution [27], [33], [39], [40]. To address the controversial stoichiometry of rhodopsin/Gt complex, we employed analytical ultracentrifugation (AUC) to determine the molecular weight of the purified complex more accurately than it is possible by size exclusion chromatography. Fitting of the equilibrium sedimentation profile (Figure 4) yielded a molecular weight of 194^+^/−1 kDa. However determination of the oligomerisation state of the RhoM257Y/Gt complex is complicated by the presence of detergents, which contribute to the molecular weight and affect the buoyancy of the particle. The size of the detergent micelle can be estimated from the excess molecular weight (ΔM) corrected for the partial specific volume difference. Using M_det_ = ΔM(1−V_p_*ρ)/(1−V_d_*ρ), where V_p_ and V_d_ are partial specific volumes of the protein and detergent, respectively, and ρ is the density of the buffer, we obtained a micelle size of 70 kDa. The expected molecular weight of the protein component of the 1∶1 complex is 124 kDa, the molecular weight of the Rho alone is 39 kDa. Taken the detergent component into account, as discussed above, this leaves the only possibility that the complex we observed has 1∶ 1 stoichiometry.

### Thermostability improvement of the complex

**Figure 5:**
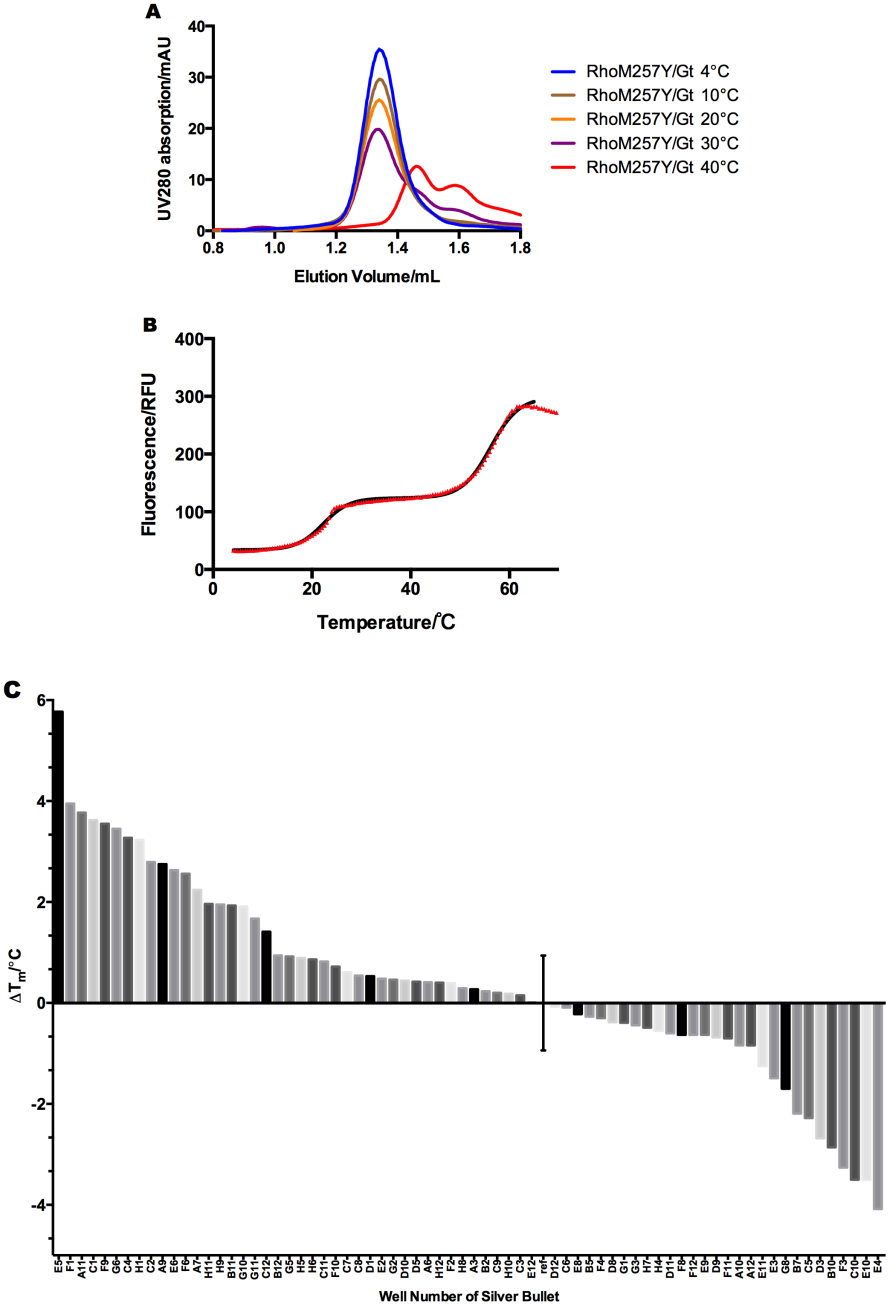
RhoM257Y/Gt complex dissociates at room temperature. **A:** analytical SEC. **B:** Fluorescent dye-assisted assay gives two transition phases arising from dissociation and unfolding of the components. Raw data (red triangles) are fitted by biphasic Boltzmann sigmoidal equation. **C:** The plot shows the stabilizing or destabilizing effect of 72 small molecule mixes from the Silver Bullet (Hampton) additive screen on the RhoM257Y/Gt complex.

Whereas the purified RhoM257Y/Gt complex exhibits full activity and resistance to wide range of pH and high salt concentrations, it readily dissociated at room temperature (Figure 5A). To be able to measure stability more accurately and in a higher throughput than by size exclusion chromatography we have established a thermo-shift assay to compare the stability of the RhoM257Y/Gt complex under various conditions. The employed fluorescence assay is based on binding of the thiol specific maleimide CPM to cysteines that become exposed during unfolding of the protein [41]. Melting curves of the RhoM257Y/Gt complex obtained with this assay showed a clear transition of the fluorescence signal at 22°C (Figure 5B) and a later transition at 56°C. Among 8 cysteines the Gαt subunit possesses, there are two cysteine residues that are supposedly protected upon complex formation, one located at the boundary between Gβγt subunit and the other one within the C-terminus. The latter one will be buried deep within the G-protein binding pocket in the rhodopsin, assuming the C-terminus of the Gαt subunit binds similarly as in the structure of the RhoM257Y with co-crystallized GaCT peptide [11]. Upon complex dissociation, these cysteines will be exposed and become available for reaction with the fluorescent dye. While the first transition is thus likely due to dissociation of the complex, the second transition likely stems from unfolding of the complex components, as both active rhodopsin and the G-protein subunits unfold between 50°C and 60°C. This interpretation of the melting curves correlates well with the data from the analytical size exclusion chromatography indicating a dissociation of the complex between 20°C and 30°C.

In a next step we used the thermo-shift assay for the search of conditions that could further stabilize the complex. In an initial screen we investigated the possibility to stabilize the complex through binding of small molecules from the Silver bullet additive screen (Hampton). This screen contains 96 different conditions each containing 2–20 small molecules that are included for their ability to stabilize intermolecular, hydrogen bonding, hydrophobic and electrostatic interactions with proteins. Colored solutions were excluded in our screen as would interfere with the fluorescence-based assay. Thermostability of the RhoM257Y/Gt complex in the presence of the remaining 72 conditions (Figure 5 C) varied with a maximal stabilizing effect of 5.8°C at a standard deviation of 0.9°C obtained from 20 measurements in the absence of additives. The best hit (E1) contained a digest of DNA and RNA with DnaseI and RnaseA, while the second best hit (F1) contained sodium pyrophosphate and sodium triphosphate, two nucleotide analogues. In both cases the observed stabilizing effect therefore is likely conferred from binding to the nucleotide-binding pocket of the Gα subunit. Because the binding of GDP or GTP leads to the dissociation of the complex, it is somewhat counter-intuitive that other nucleotides or phosphates stabilize it. However, the products of DNA and RNA digestion are complex mixtures of di-, tri- and oligo- monophosphate nucleotides and probably cannot induce the same conformational changes in Gα as GDP or GTP. On the other hand, they may stabilize the local structure of the binding pocket without causing dissociation of the complex.

### Preparation and characterization of RhoM257Y/Gi complex

**Figure 6:**
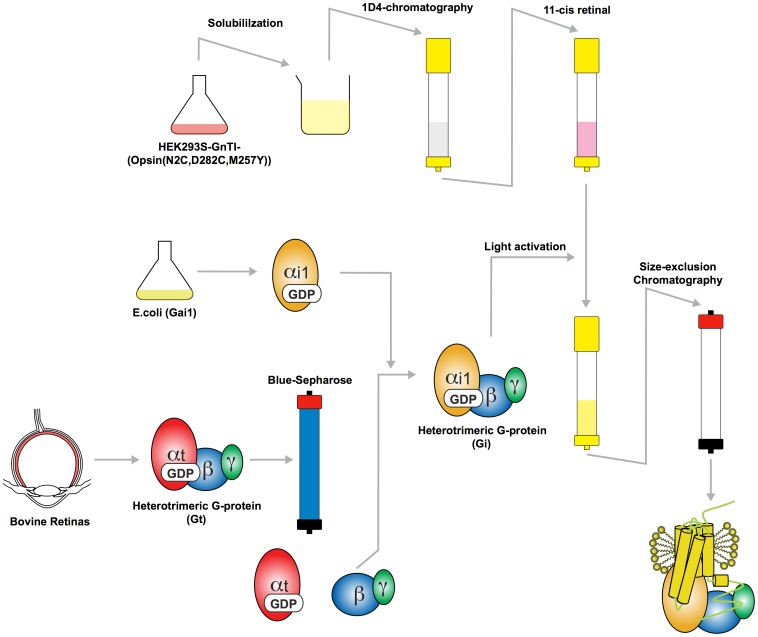
Preparation diagram of RhoM257Y/Gi complex. Heterotrimeric Gi protein is prepared by combining recombinant Gαi1 and native Gβγt separated from heterotrimeric Gt protein. RhoM257Y/Gi is formed on the 1D4-sepharose matrix and further purified by a size exclusion chromatography in the same way as RhoM257Y/Gt.

Bovine rhodopsin couples with Gt protein in the native photoreceptor, but it has been shown to activate also Gi type G-protein *in vitro* [42]. In the trial to obtain a stable rhodopsin/G-protein complex, we prepared RhoM257Y/Gi instead of RhoM257Y/Gt and investigated the thermostability using fluorescence-detection size-exclusion chromatography-based thermostability assay (FSEC-TS) [43]. Unlike Gαt, Gαi1 can be highly expressed recombinantly in bacterial cells and purified in large quantity. After reconstitution of Gi heterotrimer by combining with Gβγt separated from native Gt heterotrimer, Gi was mixed with the ground state RhoM257Y followed by photo-isomerization to form RhoM257Y/Gi in the same way as RhoM257Y/Gt (Figure 6).

**Figure 7:**
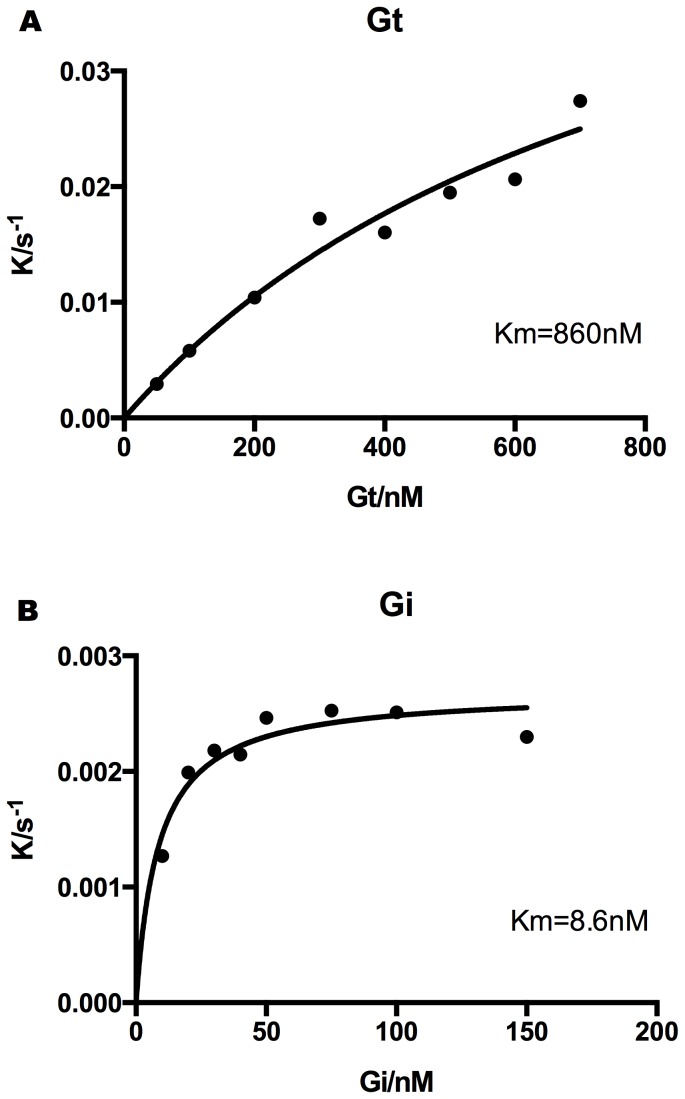
Michaelis-Menten characterization of the Gt and Gi activation by Rhodopsin using Trp fluorescence assay. The Michaelis-Menten constant (*K*_m_) of Gt to the photoactive rhodopsin is 860 nM in DDM detergent solution (**A**), while that of Gi is 8.6 nM (**B**), nearly 100 times higher than that of Gt.

**Figure 8:**
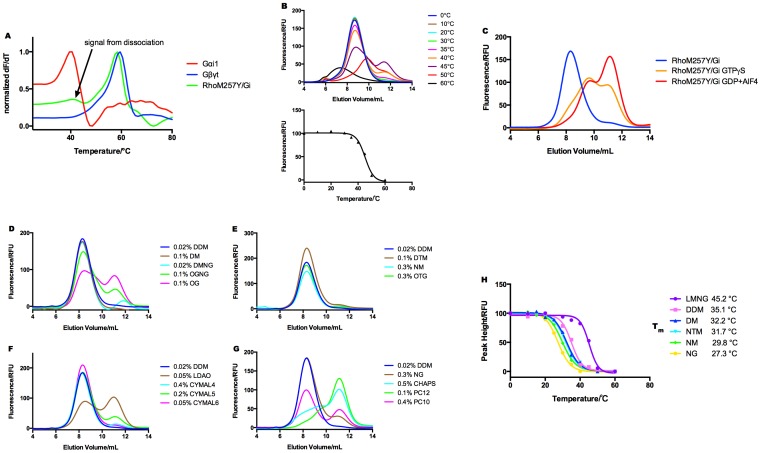
RhoM257Y/Gi is active and shows higher stability than RhoM257Y/Gt. **A:** fluorescent dye (CPM) assisted thermal denaturation assay and **B:** FSEC-TS assay. **C:** The Gi in RhoM257Y/Gi complex adopts active conformation showing dissociation upon incubating with GTPgS or GDP and AlF4. **D,E,F,G:** RhoM257Y/Gi complex is resistant to wide variety of detergents. **H:** Thermostability analysis of RhoM257Y/Gi by FSEC-TS in selected detergents.

The apparent affinities of Gt and Gi to photo-activated rhodopsin were measured by an enzymatic reaction based assay [33]. The initial rate of G-protein activation was determined by monitoring the rise of intrinsic tryptophan fluorescence. Plotting the initial rate constants against the titrated G-protein concentrations in the reaction gave a Michaelis-Menten type hyperbolic function and K_m_ value obtained after curve fitting represents apparent affinity. While K_m_ of Gt is >800 nM (Figure 7A), that of Gi is <10 nM (Figure 7B), significantly higher apparent affinity. Consistent with the higher affinity of Gi, purified RhoM257Y/Gi gave the dissociation temperature (T_d_) of 45°C in FSEC-TS and 40.5°C in CPM thermo-stability assay, respectively (Figure 8A, B).

RhoM257Y/Gi holds the activity to specifically bind GTP analogues and dissociate, which was demonstrated by analytical SEC after incubating the complex with various nucleotides (Figure 8C).

Resistance to detergents is another critical factor for the crystallization of membrane proteins as it allows the screening of a larger crystallization space. We therefore tested detergent resistance of the purified RhoM257Y/Gi complex by diluting it into a range of detergents. After incubation for 30 minutes the diluted complexes were analyzed for their structural integrity by fluorescence-detection size exclusion chromatography (Figure 8D,E,F,G). While dissociated in relatively harsh detergents, the complex survived in a number of detergents and was further investigated for the T_d_ in selected ones. As expected, RhoM257Y/Gi showed T_d_ ranging from 27°C in nonyl-glucoside to 45°C in LMNG (Figure 8H).

## Discussion

The earliest attempts at the isolation of a rhodopsin/Gt complex in soluble form were based on immobilizing rhodopsin on a ConA-Sepharose column. While this approach did not allow purification of the complex, it could be shown that Gt bound to the column and could be specifically eluted by addition of GTPγS [31]. More recently several groups have undertaken purification of the complex in detergent solution [33], [44]. These efforts have used native rhodopsin isolated from bovine retina and employed sucrose density gradient centrifugation or size exclusion chromatography to separate the activated complex from unbound material. In an earlier study [27], we introduced two modifications to achieve preparation and purification of an activated complex. First, we used immunoaffinity chromatography with a rhodopsin-specific 1D4 antibody for purification of the complex. Second, we used recombinant rhodopsin in order to take advantage of constitutively active mutations in the protein that supposedly enhances formation and stability of the activated complex. The activated complex could be formed and purified readily beginning with crude extracts from stably transfected HEK293 cells that were applied directly to the 1D4-immunoaffinity resin. Immobilization of opsin on the matrix was followed by incubation with retinal and Gt protein to form an active complex, which could be isolated in purified form by specifically eluting from the matrix with the 1D4 peptide. Although the isolated complex displayed the expected functional characteristics, it was proved unsuitable for routine large-scale purifications due to the low expression levels of the E113Q mutant and an overall low stability of the complex. In the present study we therefore utilized the RhoM257Y that leads to similar levels of constitutive activity as the E113Q mutation through a specific stabilization of the G-protein binding site [11]. Introduction of the enzyme apyrase dramatically increased the efficiency of the complex formation by preventing re-binding of GDP, which otherwise leads to the dissociation of the complex. An additional size exclusion chromatography improved homogeneity of the complex and furthermore allows for an easy exchange of detergents in crystallization screening. After successive optimization yields of the RhoM257Y/Gt complex reached 6–9 mg, sufficient amount for crystallization screens. The purified complex contained stoichiometric amounts of the retinal ligand, covalently bound by means of an unprotonated Schiff base for over a month. Incubation with GTPγS caused dissociation of the complex demonstrating that the isolated complex is functionally active. Analytical ultracentrifugation analysis of the isolated complex demonstrated the component stoichiometry of RhoM257Y and the Gt protein to be at a 1∶1 molar ratio. While this result disagrees with a recent electron microscopic and spectroscopic characterization of a native Rho/Gt complex [38], [45], it agrees well with previous reports for the complex with native rhodopsin purified by size-exclusion chromatography in DDM solution [27], [33] and that of the β2AR/Gs complex [16]. Our data also corroborate the stoichiometry observed for the rhodopsin/Gt complex in nanodiscs [39]. Based on stoichimetry, ligand-bound state, and nucleotide binding activity we conclude that the purified RhoM257Y/Gt complex consists of the active species MetaII bound to the empty-pocket state of the Gt in a 1∶1 ratio.

One factor that has long prevented the crystallization of membrane proteins and particularly GPCRs is their low stability in detergent solution. Our analysis demonstrates that the complex consisting of the thermostabilized, constitutively active RhoM257Y and the native Gt can be stored for over a few weeks at 4°C in detergent solution, long enough for a successful crystallization. Therefore, our studies suggest the purified RhoM257Y/Gt complex is suitable for crystallization by vapor diffusion at 4°C. However, dissociation of the RhoM257Y/Gt complex at temperatures above 20°C limits the chances for success in crystallization in lipidic cubic phases (LCP). This technique so far delivered most GPCR structures. In order to further stabilize the complex, we implemented a fluorescence-based high-throughput thermal stability assay that requires protein in the low microgram range and delivers an accuracy of 1°C to measure the influence of chemical additives to complex stability. Among the 72 conditions screened from the Silver Bullet crystallization additive screen (Hampton Research), 14 conditions (containing 2–20 small molecules each) increased the thermal stability by more than two standard deviations with a maximal stabilizing effect of 5.8°C. These initial hits contained nucleotide analogues that stabilize the Gt protein possibly by specific binding to the nucleotide binding pocket, as well as small molecules like benzamidine, glycerol, sugars or individual amino acids that stabilize the complex by less specific mechanisms. Interestingly similar nucleotide analogues have been used as additives in the recent crystallization of the β2AR-Gs protein complex [16].

We also found that Gi, another G-protein subtype, forms higher affinity and far more thermostable complex with rhodopsin. The reason for the higher affinity of Gi to rhodopsin is not immediately obvious. One possibility is that the concentration of both rhodopsin and Gt in rod cells is high in comparison to Gi-coupled receptors and Gi in other cell types and the high affinity is not required. Moreover, the off-rate of activated Gt from the rhodopsin may have to be high (resulting in the decreased affinity) in order to improve the overall speed of the visual signaling cascade. From the practical perspective, Gt and Gi are highly homologous and the RhoM257Y/Gi complex can be transferred into a wide range of detergents including different members of the maltoside and cymal classes that are among the most commonly used for the structure determination of membrane proteins.

Although additives and substitution of the G-protein stabilize the rhodopsin/G-protein complex, the overall instability and, more particularly, the flexibility of the alpha-helical domain of the Gα protein might be still high to hamper the crystallization [46], [47]. Consequently, further efforts would be needed on the stabilization of the complex through conformation-specific antibodies or nanobodies [16], [48], [49], [50], or engineering G-protein in order to increase the chances of successful crystallization.

## Materials and Methods

### Expression of constitutively active rhodopsin at bioreactor scale

The N2C,M257Y,D282C rhodopsin mutant (RhoM257Y) was expressed in stably transfected HEK293S-GnT1− cells [51] constructed as described previously [11]. In contrast to our previous reports we expressed the RhoM257Y in fully instrumented 20 L stirred-tank bioreactors (Sartorius, Germany) under controlled conditions (120 rpm, pH 7.2, pO2 30% air saturation) to be able to produce sufficient protein for crystallization screening. Typically, 5 days after inoculation from shake flask cultures the cell density reached 4–5×10E6 viable cells/mL (PEM medium (Life Technologies, USA) with 5% FBS, 4 mM glutamine, G418 and blasticidin as selection markers). Protein expression was induced by adding tetracycline in 500 mL of PEM medium (final concentration of 2 µg/mL tetracycline). 800 mL concentrated feeding solution (Roche, proprietary composition) was added to avoid nutrient limitations. 48 h post-induction the culture was supplemented with sodium butyrate (final concentration of 3 mM butyrate) and with additional feeding solution (400 mL). Cells were harvested 72 h post-induction by centrifugation at 3,000 g for 10 minutes at 4°C (1 L beakers, Beckman). Cell pellets (700–900 g in total) were washed once in PBS and frozen in 50 mL Falcon tubes until purification of the complex.

### Purification of the Gt protein and separation of Gβγt subunit

The Gt protein was purified from bovine retina essentially as described previously [52]. Frozen dark adapted bovine retinas, purchased from W L Lawson Company (Omaha, NE), were exposed to the room light at 4°C for overnight to form rhodopsin/Gt in the rod photoreceptor cell outer segments (ROS). Following isotonic and hypotonic washes, 40 µM GTP was added to release Gt from the purified ROS membranes. Gt was then separated from ROS by centrifugation, filtered through a 0.22 µm membrane (Steriflip from Millipore Corp., Billerica, MA), concentrated, and dialyzed against 10 mM Tris, pH 7.4, containing 2 mM MgCl2, 1 mM DTT, and 50% glycerol for storage at −20°C. Gβγt was separated from Gt heterotrimer by Blue Sepharose 6 Fast Flow (GE Healthcare) as described previously [53], flash frozen and stored at −20°C until use.

### Expression and purification of recombinant Gαi1

Human G protein alpha-subunit (Gαi1) was cloned into the pJ411 vector (DNA 2.0), incorporating an N-terminal 10-histidine tag followed by a TEV cleavage site. The sequenced plasmid was transformed into *E.coli* BL21 (DE3) strain. The bacterial cells were grown at 37°C. When OD_600_ reached 0.6, the protein expression was induced by addition of 1 mM isopropyl-β-D-thiogalactopyranoside (IPTG) and the cells were further incubated for 20 hours at 20°C. After centrifugal harvesting, the cell pellets were resuspended in buffer A (25 mM Tris-HCl, pH 7.4, 0.5 M NaCl, 50 mM imidazole, 10% glycerol) and disrupted by sonication. The supernatant was loaded into 5 ml His-Trap FF crude column (GE Healthcare). The unbound protein was washed with buffer A and eluted with buffer B (25 mM Tris-HCl pH 7.4, 0.5 M imidazole, 0.5 M NaCl and 10% glycerol). After the cleavage of the histidine tag, the cleaved Gαi1 protein was further purified by a size exclusion chromatography (HiLoad Superdex 200, GE Healthcare).

### Purification of RhoM257Y/Gt and RhoM257Y/Gi complex

Purification of RhoM257Y/Gt complex was performed essentially in the same way as described previously [27] using N2C/D282C/M257Y mutant bovine opsin (OpsinM257Y) instead of N2C/D282C/E113Q mutant. Briefly, the whole HEK293S-GnTI^−^ cells, stably expressing OpsinM257Y, are solubilized in β-dodecyl-D-n-maltoside (DDM). After separating the supernatant, OpsinM257Y was first immobilized onto the 1D4-antibody immunoaffinity sepharose and reconstituted with 11-cis retinal to form the ground state RhoM257Y. The ground state RhoM257Y was mixed with purified Gt and irradiated for 10 to 15 minutes through 495 nm long-pass filter, converting the inverse agonist 11-cis retinal to the full agonist all-trans retinal and forming RhoM257Y/Gt complex on the sepharose resin. The resulting RhoM257Y/Gt complex was detergent-exchanged from 0.02% DDM to 0.02% lauryl-maltose neopentyl glycol(LMNG) and eluted from the resin by incubating with 1D4-elution peptide (TETSQVAPA). The eluent was further purified by size-exclusion chromatography. Adding 25 mU/ml Apyrase (New England Biolabs) during the light activation improved the efficiency of the RhoM257Y/Gt complex formation by preventing re-binding of the GDP that was released from Gαt after the binding of Gt to the active RhoM257Y [16]. Heterotrimeric Gi protein was formed by mixing purified Gαi1 and Gβγt subunits at equimolar ratio for 30 min on ice, and used for the formation of RhoM257Y/Gi complex in the same way as RhoM257Y/Gt complex.

### Acid denaturation assay

UV/Vis absorption spectra were measured by using a UV_2401PC spectrophotometer (Shimadzu). Spectra of the intact complex were measured by diluting 0.2 ul of purified RhoM257Y/Gt complex (60 mg/ml) into 100 ul of buffer (100 mM NaCl, 10 mM Hepes pH 7.5 and 0.02% LMNG). Spectra of the acid denaturated complex were measured after adding 2 ul of 25%H_2_SO_4_ and 3.5 ul of 20% Sodium dodecyl sulfate to make the pH around 1.9 to denature the protein and protonate retinilydene Schiff bases.

### G protein Activation Assay

The G protein activation was measured by monitoring the change in intrinsic tryptophan fluorescence in the Gα subunit upon exchange of GDP to GTPγS. All measurements were performed by using Varian Cary Eclipse fluorescence spectrophotometer with settings of λ_ex_ = 295 nm and λ_em_ = 340 nm. The assays were carried out with 1 or 30 nM native rhodopsin (purified from bovine retina), 0.01% DDM, and buffer C (50 mM Bis-Tris, pH 7.3, 130 mM NaCl, 1 mM MgCl_2_, 1 mM DTT) in a final volume 1 ml (10×2 mm cuvette with stirring bar) at 20 degree. The hetetrotrimeric Gi protein was reconstituted by mixing equimolar ratio of the recombinant Gαi1 and native Gβγt (purified from bovine retina) on ice for 30 min. The native Gt was prepared from bovine retina. After forming the protein complex of R*·G by irradiation to the orange light (>495 nm), the basic fluorescence of R*·G was monitored for 5 min followed by the addition of 10 µM GTPγS. The fluorescence intensity was continuously recorded for 1 hr. The entire set of experiments was repeated with increasing concentrations of Gi or Gt. The initial G protein activation rate was determined by fitting the fluorescence intensity to an exponential association curve y = y0+a[1−exp(−*k*_r_′*t*)] using Origin 8.5, where *k*_r_′ is the apparent rate constant and *t* is the time in seconds. Apparent rate constant (*k*_r_′) of the initial fluorescence increase was plotted against G protein concentrations. The data were fitted by Michaelis-Menten equation: *k*_r_′ = (*V_max_*·[G])/(*K_m_*+[G]).

### CPM fluorescence assisted Thermo-stability assay

Thermal dissociation was monitored by the formation of the thiol specific malemaide fluorochrome CPM (N-[4-(7-diethylamino-4-methyl-3-coumarinyl)phenyl]malemeide) adduct [41] attached to the protected cystein residue between Gαt or Gαi1, and Gβγt.

For RhoM257Y/Gt, thermo-stability assays were performed using Eclipse fluorimeter (Varian) equipped with a multisample holder. 2 µl of purified RhoM257Y/Gt (1 mg/ml) was diluted into 98 µl ice cold buffer (100 mM NaCl, 10 mM Hepes pH 7.5 and 0.02% LMNG). Immediately before the measurement, CPM (3 mg/ml in DMSO) was diluted 1∶30 into buffer and 10 µl of the diluted CPM was added to the reaction mix. Cuvettes were placed into the fluorimeter and fluorescence intensity (λ_ex_: 387 nm, λ_em_:464 nm) was monitored while ramping temperature from 4°C to 90°C at the rate of 2°C/min. The resulting curve was fitted using a sigmoidal Boltzmann equation to obtain Td_50_ values. Comparison of thermo-stability in the presence of additives was performed by supplementing the reaction mix with 10 µl of each condition from the Silver Bullet additive screen. For each set of comparisons a control without additives was included and used to calculate ΔTd values. The standard deviation from 20 measurements of the RhoM257Y/Gt complex was 0.94°C.

For RhoM257Y/Gi, thermo-stability assay was performed using Rotor GeneQ (Qiagen). 5 ug of purified RhoM257Y/Gi was diluted into 120 ul ice cold buffer (100 mM NaCl, 10 mM Hepes pH 7.5 and 0.01%LMNG). 10 ul of the freshly prepared 40∶1 dilution of CPM into measuring buffer was added immediately before the measurement. We prepare CPM stock at 3 mg/ml in DMSO. Fluorescence intensity (λ_ex_: 365 nm, λ_em_: 460 nm) was monitored while ramping temperature from 25°C to 90°C at the rate of 4°C/min. The resulting curves were analyzed by the Rotor GeneQ package software.

### Analytical size-exclusion chromatography

For RhoM257Y/Gt, 18 ug of purified complex in 100 ul of buffer composed of 100 mM NaCl, 10 mM Tris pH 7.5, and 0.02% LMNG or other salt or buffer conditions mentioned in the results was loaded onto superdex 200 PC 3.2/30 (GE Healthcare) equilibrated with 100 mM NaCl, 10 mM Tris pH 7.4, 20% glycerol, and 0.02% LMNG and run at 0.05 ml/min. The elution profile was monitored by the absorption of λ: 280 nm. For RhoM257Y/Gi, 1 ul of complex (1 mg/ml) was diluted into 100 ul of buffer composed of 100 mM NaCl, 10 mM Hepes pH 7.5, and 0.01% was loaded onto superdex 200 packed in a Tricorn 10/200 column (GE Healthcare) equilibrated with 100 mN NaCl, 10 mM Hepes pH 7.5, 0.01% LMNG. The elution profile was monitored by protein-intrinsic fluorescence with λ_ex_: 280 nm, λ_em_: 340 nm. For detergent resistance test, 1 ul of RhoM257Y/Gi (1 mg/ml) purified in DDM was diluted and incubated for 30 minutes in 100 ul of buffer composed of 100 mM NaCl, 10 mM Hepes pH 7.5, and each detergent followed by FSEC.

For FSEC-TS, 1 ug of RhoM257Y/Gi in 100 ul of the buffer was incubated at 4°C to 60°C for 30 minutes, ice cooled for 5 minutes, and then centrifuged at 9,000 g for 5 minutes. The peak heights were normalized and then fit to a sigmoidal dose-response curve to obtain T_d_ values.

### Analytical ultracentrifugation

Equilibrium sedimentation experiments were done in a Beckman Optima-XLI instrument at 4°C, using An-60Ti rotor and 6-sector centrepieces. Data were collected at 11k, 15k and 20k rpm and analyzed using the UltraSpin software (D. Veprintsev). Partial specific volume was calculated from the protein sequence to be 0.737 using Sedenterp software (J. Philo). Buffer conditions were 20 mM KPi, 100 mM NaCl, 0.02% MNG-3. Partial specific volume of LMNG was assumed to be similar to DDM (0.82 ml/gr).
